# Accelerated hypofractionated radiation therapy compared to conventionally fractionated radiation therapy for the treatment of inoperable non-small cell lung cancer

**DOI:** 10.1186/1748-717X-7-33

**Published:** 2012-03-15

**Authors:** Arya Amini, Steven H Lin, Caimiao Wei, Pamela Allen, James D Cox, Ritsuko Komaki

**Affiliations:** 1 Department of Radiation Oncology, The University of Texas MD Anderson Cancer Center, Houston, TX, USA; 2 Department of Biostatistics, The University of Texas MD Anderson Cancer Center, Houston, TX, USA; 3 UC Irvine School of Medicine, Irvine, CA, USA; 4 University of Texas MD Anderson, Unit 97, 1515 Holcombe Blvd., Houston, TX 77030, USA

**Keywords:** Accelerated radiotherapy, Non-small cell lung cancer, Hypofractionated

## Abstract

**Background:**

While conventionally fractionated radiation therapy alone is an acceptable option for poor prognostic patients with unresectable stage III NSCLC, we hypothesized that accelerated hypofractionated radiotherapy will have similar efficacy without increasing toxicity.

**Methods:**

This is a retrospective analysis of 300 patients diagnosed with stage III NSCLC treated between 1993 and 2009. Patients included in the study were medically or surgically inoperable, were free of metastatic disease at initial workup and did not receive concurrent chemotherapy. Patients were categorized into three groups. Group 1 received 45 Gy in 15 fractions over 3 weeks (Accelerated Radiotherapy (ACRT)) while group 2 received 60-63 Gy (Standard Radiation Therapy 1 (STRT1)) and group 3 received > 63 Gy (Standard Radiation Therapy (STRT2)).

**Results:**

There were 119 (39.7%) patients in the ACRT group, 90 (30.0%) in STRT1 and 91 (30.3%) in STRT2. More patients in the ACRT group had KPS ≤ 60 (p < 0.001), more commonly presented with weight loss > 5% (p = 0.002), and had stage 3B disease (p < 0.001). After adjusting for clinical variables, there were no differences in the radiation groups in terms of the patterns of local or distant tumor control or overall survival. Some benefit in relapse free survival was seen in the STRT1 group as compared to ACRT (HR = 0.65, p = 0.011). Acute toxicity profiles in the ACRT were significantly lower for grade ≥ 2 radiation dermatitis (p = 0.002), nausea/vomiting (p = 0.022), and weight loss during treatment (p = 0.020).

**Conclusions:**

Despite the limitations of a retrospective analysis, our experience of accelerated hypofractionated radiation therapy with 45 Gy in 15 fractions appears to be an acceptable treatment option for poor performance status patients with stage III inoperable tumors. Such a treatment regimen (or higher doses in 15 fractions) should be prospectively evaluated using modern radiation technologies with the addition of sequential high dose chemotherapy in stage III NSCLC.

## Background

Worldwide, lung cancer is the leading cause of cancer-related deaths with approximately 1.4 million deaths annually [1]. Approximately 80% of these cases are from non-small cell lung cancer (NSCLC). The current standard treatment approach for medically or surgically inoperable NSCLC is once-daily radiation treatments to 60 Gy at 2 Gy per fraction, established by Radiation Therapy Oncology Group (RTOG) 7,310 trial[2]. It has been proven subsequently that disease outcome can be further improved by the addition of concurrent chemotherapy as demonstrated recently by the publication of RTOG 9410[3]. However, for more elderly patients with poor prognostic factors including initial weight loss ≥ 5%, Karnofsky performance status (KPS) scores < 70, and additional health comorbidities, the standard regimens become very difficult to determine. Several studies have identified initial performance status and weight loss to be important prognostic factors in predicting survival in NSCLC [4]. As such, these factors become important in assessing treatment options for these patients.

More than half of patients who are diagnosed with NSCLC are over the age of 65. Concurrent chemoradiation given for six weeks may be difficult for these patients to tolerate, especially those presenting with poor performance status and significant weight loss from their cancers. In addition, many of these patients present with a host of co-morbid illnesses, making clinicians less inclined to treat with full course radiation and concurrent chemotherapy. Several studies have demonstrated significant rates of increased toxicity and overall poorer survival in elderly patients with poor prognostic factors, raising the need for alternative treatment management in patients who cannot tolerate standard therapy [5].

Currently, patients with poor prognostic factors are commonly treated with radiation therapy alone since concurrent chemotherapy is usually not tolerated. A study by Nguyen et al. [6] performed at our institution reported our initial experience in patients treated with accelerated hypofractionated radiotherapy (ACRT). Patients were separated into two treatment categories based on their performance status and initial % weight loss. Patients with a KPS score ≥ 70 and weight loss ≤ 5% were treated conventionally (60-66 Gy at 2 Gy per fraction, N = 29). Patients with poor performance status and weight loss > 5% were treated with ACRT (45 Gy at 3 Gy per fraction, N = 26). The study showed no statistically significant difference in treatment response rates, locoregional control and overall survival between the two treatment arms. Treatment toxicity was also not found to be different between the two groups. With this initial experience, 45 Gy became an option at our institution for patients who could not tolerate the standard regimen.

For this study, we report our updated experience in stage III NSCLC patients treated with 45 Gy in 15 fractions and compared to patients who received 60 Gy or higher conventionally fractionated radiation therapy. We investigated the efficacy, toxicity, recurrence rate and survival rates of the ACRT regimen compared to the more conventionally fractionation radiotherapy.

## Methods

### Patient characteristics

We reviewed treatment records of 2,657 patients treated for lung cancer at MD Anderson Cancer Center between 1993 and 2009. Out of these patients, 1,982 individuals received conventional fractionation (≥ 60 Gy) and 655 patients received accelerated radiation therapy (45 Gy). Staging was based on the American Joint Committee Classification (AJCC) 7th edition criteria. We confined our analysis to patients who had histologically confirmed stage IIIA-IIIB NSCLC that were not previously treated and did not receive concurrent chemotherapy. Patients with small cell lung cancer, thymic tumors and carcinoid were excluded. In all, 300 patients formed the study cohort. All patients in the study cohort were able to complete treatment. KPS scores prior to treatment were recorded based on physicians' notes. Initial percent weight loss from the disease was recorded based on patient reports. Information of tumor size was gathered from the imaging reports. This post hoc analysis was approved by the institutional review board of MD Anderson.

Of the 300 patients identified for the study, 119 patients received accelerated radiotherapy (ACRT), at 45 Gy in 15 fractions over 3 weeks, 90 patients received standard radiation therapy at 60-63 Gy in 6 weeks (STRT1), and 91 individuals received standard radiation therapy > 63 Gy in 6 weeks (STRT2). All patients were Computed Tomography (CT) simulated and immobilized using the standard techniques at our institution.

### Treatment and outcomes assessment

CT and Positron Emission Tomography (PET) were primarily used to evaluate for disease recurrences following treatment. Scanned documents and imaging reports from outside facilities were also used to evaluate for patterns of failure. Pathology reports were used when available. Both local and distant failures were recorded for all patients. Dates of death were found by scanned follow up letters in patients' medical records and the social security death index.

Radiation treatment toxicity was collected using progress notes as well as longer-term follow up notes to assess for radiation-induced side effects. The Common Terminology Criteria for Adverse Events (CTCAE) v4.03 grading system was used to assign a numerical number to clinicians' notes.

### Statistical analysis

Chi-squared test (when expected counts ≥ 5 for all cells of the cross table) or Fisher's exact tests (when expected counts < 5 for some cells) were used to assess the associations between categorical variables and radiation therapy groups. Wilcoxon tests were used to assess the association between age and radiation treatment groups. Overall survival (OS) interval was calculated from the date of diagnosis to the date of death or last known date that the patient was alive. Recurrence Free Survival (RFS) interval was calculated from the date of diagnosis to the date of relapse, or the date death or last known date whichever occurred first. Deaths without any failure were considered competing risk events. OS and RFS were first examined by the method of Kaplan and Meier. The hazard ratios of radiation therapy groups were computed by a Cox model proportional hazards model [7], adjusted for induction chemotherapy and initial weight loss, as well as other variables selected by the backward selection procedure. The following variables were examined: age at diagnosis, gender, KPS, grade, adjuvant chemotherapy, N-stage, T-stage, and overall clinical stage. Variables were selected by the backward selection with an adjusted p-value not greater than 0.1.

Cumulative incidence estimates of disease recurrence were estimated by subdistribution analysis of competing risks [8]. Competing risk regression models [8] were used to assess the association between failures and radiation therapy groups. For locoregional control, we adjusted for age. For distant recurrence, we adjusted for age, and histology.

## Results

### Patient characteristics

Among the 300 patients, 159 (53.0%) were male, 141 (47.0%) were female. Median age at diagnosis was 69.5 years (range 41-100 years). As of the most recent follow-up, there were 254 (84.7%) deaths. There were 165 (55%) patients who experienced one or both local (n = 77) and distant failures (n = 119). Overall, 188 patients (62.7%) died without locoregional failure, 148 (49.3%) died without distant failure, and 108 individuals (36.0%) died without evidence of disease recurrence.

Patient and tumor characteristics are summarized in Table 1. Patients in the ACRT group more commonly had KPS scores ≤ 70 and an initial weight loss ≥ 5%. These patients more often had stage 3B vs. 3A disease. Patients receiving 60-63 Gy presented with a similar median age compared to the ACRT cohort. In this group both genders were more equally represented, with KPS scores ranging between 70 and 80; they more often presented with weight loss < 5%. These patients more commonly presented with stage 3A disease and had statistically significant higher rate of induction chemotherapy. Patients receiving > 63 Gy were older, had KPS scores ≥ 80, weight loss < 5%, and with stage 3A and 3B more evenly distributed (Table 1).

**Table 1:** Patient/Treatment Characteristics

Variable		ACRT	STRT1	STRT2	Total	*p*-value
		(45 Gy)	(60-63 Gy)	(> 63 Gy)		
		n = 119	n = 90	n = 91		
Age	median(range)	68(41,100)	67(44,88)	73(47,95)	69.5(41,100)	< 0.001
						
Gender	Female	48(40.3%)	43(47.8%)	50(54.9%)	141(47.0%)	0.108
	Male	71(59.7%)	47(52.2%)	41(45.1%)	159(53.0%)	
						
Smoking Status	Never	8(6.8%)	2(2.2%)	4(4.4%)	14(4.7%)	0.224
	Quit	70(59.8%)	45(50.6%)	53(58.2%)	168(56.6%)	
	Current	39(33.3%)	42(47.2%)	34(37.4%)	115(38.7%)	
						
Karnofsky Performance Status score	90	3(2.5%)	5(5.6%)	6(6.6%)	14(4.7%)	< 0.001
	80	35(29.4%)	58(64.4%)	40(44.0%)	133(44.3%)	
	70	47(39.5%)	23(25.6%)	38(41.8%)	108(36.0%)	
	≤ 60	34(28.6%)	4(4.4%)	7(7.7%)	45(15.0%)	
						
Presenting Weight	No	56(51.4%)	67(75.3%)	61(67.8%)	184(63.9%)	0.002
loss ≥ 5%	Yes	53(48.6%)	22(24.7%)	29(32.2%)	104(36.1%)	
						
Tumor Stage	IIIA	37(31.1%)	50(55.6%)	57(62.6%)	144(48.0%)	< 0.001
	IIIB	82(68.9%)	40(44.4%)	34(37.4%)	156(52.0%)	
						
Tumor Histology	Adenocarcinoma	37(31.4%)	31(35.2%)	41(45.6%)	109(36.8%)	0.142
	Squamous	42(35.6%)	34(38.6%)	32(35.6%)	108(36.5%)	
	NSC-NOS	39(33.1%)	23(26.1%)	17(18.9%)	79(26.6%)	
						
Tumor Grade	Well	3(2.5%)	0(0%)	4(4.4%)	7(2.3%)	0.069
	Moderate	11(9.2%)	12(13.3%)	10(11.0%)	33(11.0%)	
	Poor	37(31.1%)	42(46.7%)	29(31.9%)	108(36.0%)	
	Unclear	68(57.1%)	36(40.0%)	48(52.7%)	152(50.7%)	
						
Tumor Size (cm)	median(range)	5(1,11.5)	5(1.5,10.5)	4.2(1,9)	5(1,11.5)	0.039
						
Induction	No	96(80.7%)	29(32.2%)	64(70.3%)	189(63.0%)	< 0.001
Chemotherapy	Yes	23(19.3%)	61(67.8%)	27(29.7%)	111(37.0%)	
						
Adjuvant	No	105(88.2%)	85(94.4%)	86(94.5%)	276(92.0%)	0.15
Chemotherapy	Yes	14(11.8%)	5(5.6%)	5(5.5%)	24(8.0%)	

ACRT: accelerated radiotherapy; STRT1: standard radiation therapy 1 (60-63 Gy); SBRT2: standard radiation therapy 2 (> 63 Gy); NSC-NOS: Non-Small Cell-Not Otherwise Specified

### Locoregional and distant recurrence rates

**Figure 1:**
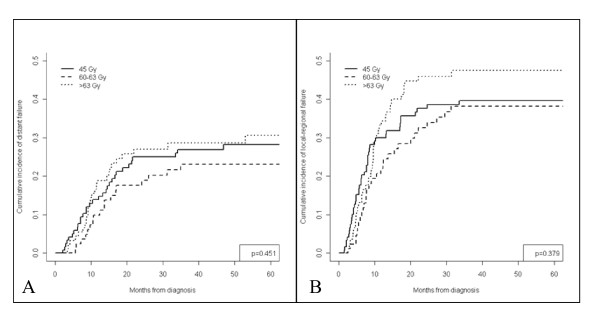
**Cumulative incidence representing rate of local-regional recurrence (Figure 1A) and distant failure (Figure 1B) for all patients based on radiation treatment groups**.

Under univariable analysis, locoregional failure and distant recurrence rates were not significant between radiation treatment (RT) groups, with similar cumulative incidence of failures for all groups (Figure 1A &1B). Age as a continuous variable was found to be a significant predictor for locoregional (Hazard Ratio [HR] 0.979, 95% Confidence Interval [CI] 0.969-0.999, p = 0.039) and distant failures (HR 0.967, 95% CI 0.967-0.999, p = 0.041). In addition, non-small cell lung cancer-not otherwise specified (nsc-nos) (HR 1.656, 95% CI 1.028-2.667, p = 0.038) and adenocarcinoma (HR 1.601, 95% CI 1.029-2.492, p = 0.037), when compared to squamous carcinoma histology, predicted for increased rates of distant failure. Under multivariable analysis, adjusting for age and histology, RT groups were again not significantly associated with locoregional or distant failures. However, younger age was significantly associated with locoregional failures, and younger age and histology (adenocarcinoma versus squamous) were independent predictors for distant metastasis (Table 2).

**Table 2:** Multivariable Analysis for Locoregional Failure and Distant Metastasis

		Locoregional Failure		Distant Metastasis	
**Variable**	**Level**	**HR (95% CI)**	**p-value (multivariable)**	**HR (95% CI)**	**p-value (multivariable)**

RT Group	STRT1 vs. ACRT	0.797 (0.455-1.398)	0.430	0.821 (0.520-1.296)	0.400
	STRT2 vs. ACRT	1.267 (0.742-2.164)	0.390	1.216 (0.792-1.867)	0.370
	STRT2 vs. STRT1	1.590 (0.890-2.840)	0.120	1.482 (0.930-2.361)	0.098
					
Age	years, continuous	0.976 (0.957-0.996)	0.021	0.982 (0.965-0.999)	0.037
					
Tumor Histology	NSC-NOS vs. Squamous	-	-	1.525 (0.933,2.494)	0.092
	Adenocarcinoma vs. Squamous	-	-	1.551 (0.996, 2.415)	0.052

HR: Hazard Ratio; CI: Confidence Interval; RT: Radiation Treatment; NSC-NOS: Non-Small Cell-Not Otherwise Specified

### Relapse free survival and overall survival

**Figure 2:**
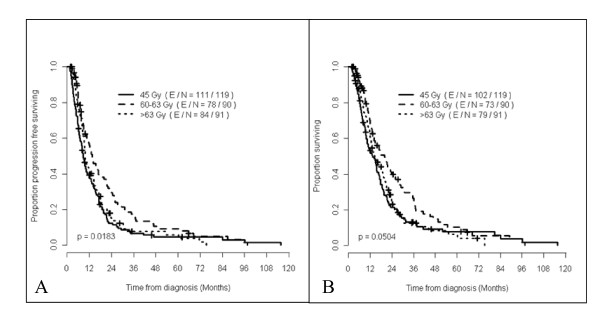
**Kaplan-Meier curves representing recurrence free survival (Figure 2A) and overall survival (Figure 2B) for all patients based on radiation treatment groups**.

Under univariable analysis, relapse free survival (RFS) was better for those in the STRT2 group (Figure 2A). After adjusting for age, induction chemotherapy, initial weight loss and tumor histology under multivariable analysis, STRT1 had improved RFS compared to ACRT1 but no difference was seen between ACRT and STRT2. Age and tumor histology were also found to be independent predictors for RFS (Table 3).

**Table 3:** Multivariate Analysis of Overall and Recurrence Free Survival

		Overall Survival		Relapse Free Survival	
**Variable**	**Level**	**HR (95% CI)**	**p-value (multivariable)**	**HR (95% CI)**	**p-value (multivariable)**

RT Group (All stages)	STRT1 vs. ACRT	0.764 (0.544-1.074)	0.121	0.649 (0.465-0.904)	0.011
	STRT2 vs. ACRT	0.885 (0.643-1.219)	0.455	0.848 (0.619-1.162)	0.304
					
Induction Chemotherapy	Yes vs. No	0.852 (0.637-1.139)	0.280	0.896 (0.675-1.189)	0.445
					
Initial Weight Loss	≥ 5% vs. < 5%	1.192 (0.909-1.562)	0.205	1.099 (0.842-1.434)	0.488
					
Age	Each 5 yr	1.122 (1.056-1.193)	< 0.001	1.091 (1.026-1.987)	0.005
					
Tumor Histology	NSC-NOS vs. Adenocarcinoma	-	-	1.428 (1.026-1.987)	0.035
	Squamous vs. Adenocarcinoma	-	-	1.180 (0.879-1.584)	0.271

HR: Hazard Ratio; CI: Confidence Interval; RT: Radiation Treatment; NSC-NOS: Non-Small Cell-Not Otherwise Specified

For overall survival, RT groups were significantly different under univariable analysis (Figure 2B), with STRT1 having the better overall survival outcome. Lower KPS was a predictor for worse OS (p = 0.005). Receipt of adjuvant chemotherapy was not a predictor for OS (p = 0.988). After adjusting for factors such as age, induction chemotherapy, and initial weight loss, RT groups did not differ significantly between ACRT compared to STRT1 and STRT2. Older age was the only independent predictor for worse OS (Table 3).

### Treatment toxicity

Treatment was generally well tolerated in all radiation therapy groups. Toxicity profiles in the 45 Gy group were significantly lower for several categories in comparison to the 60-63 Gy and > 63 Gy groups (Table 4). Grade 2 and greater radiation dermatitis, nausea and vomiting, and weight loss during treatment were all significantly less in the ACRT group. Pneumonitis was the most common toxicity found in all three groups, though about 10% less in the ACRT treatment arm. No differences were appreciated in rates of pneumonitis, esophagitis or dysphagia.

**Table 4:** Toxicity Distribution

Toxicity	Grade	ACRT	STRT1	STRT2	Total	*p*-value
		(45 Gy)	(60-63 Gy)	(> 63 Gy)		
		n = 154	n = 99	n = 239		
Esophagitis	0-1	107(89.9%)	80(88.9%)	82(90.1%)	269(89.7%)	0.958
	≥ 2	12(10.1%)	10(11.1%)	9(9.9%)	31(10.3%)	
						
Dysphagia	0-1	110(92.4%)	75(83.3%)	80(87.9%)	265(88.3%)	0.126
	≥ 2	9(7.6%)	15(16.7%)	11(12.1%)	35(11.7%)	
						
Pneumonitis	0-1	105(88.2%)	71(78.9%)	71(78.0%)	247(82.3%)	0.093
	≥ 2	14(11.8%)	19(21.1%)	20(22.0%)	53(17.7%)	
						
Radiation Dermatitis	0-1	117(98.3%)	79(87.8%)	78(85.7%)	274(91.3%)	0.002
	≥ 2	2(1.7%)	11(12.2%)	13(14.3%)	26(8.7%)	
						
Nausea/Vomiting	0-1	115(96.6%)	79(87.8%)	87(95.6%)	281(93.7%)	0.022
	≥ 2	4(3.4%)	11(12.2%)	13(14.3%)	26(8.7%)	
						
Weight loss	0-1	115(96.6%)	81(90.0%)	90(98.9%)	286(95.3%)	0.020
	≥ 2	4(3.4%)	9(10.0%)	1(1.1%)	14(4.7%)	

## Discussion

Since the original study by Nguyen et al. [6], our institution has been using 45 Gy in 15 fractions for unresectable NSCLC who otherwise cannot tolerate the conventional regimen. In that original report, and presently in this updated study with a much larger study cohort, patients receiving accelerated radiotherapy had comparable local and distant recurrence rates with no difference in overall survival compared to conventionally fractionated radiotherapy for all stages combined, even though these patients initially presented with worse prognostic factors. Our study showed no difference in cumulative incidence of locoregional or distant failures between the RT groups in the presence of competing factors. There was some difference in relapse free survival in the 60-63 Gy arm with no difference in overall survival after adjusting for other variables. Lastly, we found treatment to be well tolerated in the ACRT cohort, even though these patients presented with worse prognostic factors (weight loss > 5%, KPS < 70). Overall, our study demonstrates that accelerated radiation therapy with 45 Gy in 15 fractions is an acceptable treatment option with comparable outcomes for patients with inoperable tumors treated with conventional radiotherapy without chemotherapy.

Radiation treatment using daily accelerated hypofractionation for the treatment of locally advanced lung cancers has been reported in other studies. Slotman et al. [9] retrospectively compared three hypofractionated schemes for the treatment of unresectable NSCLC (stage IIIA-IV) (40 Gy split course, 30-32 Gy in 6 fractions, or 24 Gy in 3 fractions) demonstrated that a split course treatment regimen of 40 Gy had improved overall survival and lower local relapse rates in stage IIIA NSCLC patients, but not in patients with stage IIIB-IV disease. Kepka et al. [10] performed a dose escalation study in which patients were initially treated with a 4 week course (21 days) at 56.7 Gy (2.7 Gy per fraction), and gradually escalated to a MTD of 60.9 Gy in 21 days (2.9 Gy per fraction). Fit patients received induction chemotherapy for 2-3 cycles. The median survival was 17 months, and the 2- and 3-year overall survival rates were 32 and 19%, respectively.

There are several biological benefits of the 45 Gy in 15 fraction treatment regimen. Applying α/β ratio of 10, this regimen has a biological equivalent dose (BED10) of 58.5 Gy. Although this is lower than the BED10 for patients treated with conventionally fractionated radiation to 60-70 Gy (72-84 Gy), our preliminary data published by Nguyen et al. [6] and this updated experience seem to indicate that control rates seem similar between the two treatment schemes. The benefit may come in the shortened treatment time, which can counteract tumor repopulation. Many studies have stressed the importance of maintaining radiation treatment duration within a shortened period, to prevent tumor repopulation, which often contains more resistant cells that are much more difficult to treat. The biological benefit of accelerated radiotherapy can best be seen as a negative influence factor against rapid tumor cell proliferation. Cox et al. [11] showed a significant difference in OS in patients with treatment delays which were noted to be more frequent in patients receiving higher total doses (≥ 69.6 Gy). Other studies looking at lung cancer patients also noted potentially worse outcomes with prolonged radiation treatment times, due in part to repopulation [12,13].

Our study is limited by its retrospective nature, and apparent imbalances in the treatment groups that are inherent in observational studies. Some of our patients had poor follow-up, and therefore disease recurrence may have been more common than reported. However, the merits of our study are that this is a review of a fairly large group of patients (> 100) of a regimen of hypofractionated accelerated radiotherapy for a group of patients that would not have otherwise tolerated a longer course of therapy. The comparison groups were patients who received > 60 Gy without concurrent chemotherapy, which we believe, despite the inherent limitations, better answered the issue of dose and removed the confounding factor of patient selection for the receipt of concurrent chemotherapy.

We believe our reported experience demonstrated the tolerability and relative effectiveness of this treatment regimen. Currently concurrent chemoradiation is the standard therapy for the management of locally advanced NSCLC, based on the recently published phase III trial RTOG 9410 [3]. This regimen improved the median survival by about 3 months over induction chemotherapy and conventionally fractionated radiotherapy. However, this treatment regimen carries substantially higher grade 3 or higher non-hematologic acute toxicities, such as esophagitis and mucositis. Limiting concurrent chemotherapy with radiotherapy would minimize this additive toxicity. Since distant metastatic disease is still the major pattern of failure for locally advanced NSCLC, treatment using a regimen of high dose systemic chemotherapy sandwiched with a short course of effective local therapy may improve disease outcomes while reducing treatment related toxicities.

Building upon our experience is the basis of an ongoing dose escalation radiotherapy trial at our institution using proton beam therapy in advanced lung cancer patients (45 Gy to 52.5 Gy to 60 Gy in 15 fractions), as well as a proposed phase II trial using sequential high dose chemotherapy and the sandwiched hypofractionated radiotherapy using the MTD (from the above dose escalation trial) in 15 fractions using protons or IMRT for locally advanced NSCLC. We believe this approach for the treatment of unresectable lung cancers can become a standard in the future, by minimizing both the toxicity of concurrent chemotherapy and the delay of long course radiotherapy. By shortening the overall treatment time and allowing full dose systemic therapy to be delivered sequentially with effective local radiotherapy may improve the distant metastatic rate and accelerated repopulation potential.

## Conclusions

Our findings suggest that accelerated radiotherapy for patients with inoperable tumors is a safe, convenient and effective treatment option. Although this study is limited by the retrospective nature of the analysis, these are hypothesis-generating results which can serve as a basis of a prospective study comparing hypofractionated regimen with conventionally fractionated radiotherapy. However, with similar rates of efficacy in high-risk individuals as seen from these results, a shortened treatment time interval will reduce overall treatment cost and improve patient convenience. We believe this treatment approach will be a viable treatment option for unresectable lung cancers in the future in combination with sequential systemic therapies.

## Competing interests

The authors declare that they have no competing interests.

## Authors' contributions

AA carried out the data collection and manuscript writing; SHL helped conceive the study, participated in the study design and coordination, helped collect data, and wrote the manuscript; CW carried out the data and statistical analysis; PA carried out the study design and data analysis; JDC participated in the study design; RK provided data and participated in the coordination and design of the study. All authors read and approved the final manuscript.

## References

[B1] Jemal A Global cancer statistics CA Cancer J Clin 2011 61 2 69 90 10.3322/caac.20107 21296855

[B2] Perez CA Stanley K Rubin P A prospective randomized study of various irradiation doses and fractionation schedules in the treatment of inoperable non-oat-cell carcinoma of the lungs. Preliminary report by the radiation therapy oncology group Cancer 1980 45 11 2744 2753 10.1002/1097-0142(19800601)45:11<2744::AID-CNCR2820451108>3.0.CO;2-U 6991092

[B3] Curran WJ Jr Sequential vs concurrent chemoradiation for stage iii non-small cell lung cancer: randomized phase III trial RTOG 9410 J Natl Cancer Inst 2011 103 19 1452 1460 10.1093/jnci/djr325 21903745 PMC3186782

[B4] Feld R Pretreatment minimal staging and prognostic factors for non-small cell lung cancer Lung Cancer 1997 17 Suppl 1 S3 S10 9213295 10.1016/s0169-5002(97)00637-5

[B5] Ngeow J Impact of comorbidities on clinical outcomes in non-small cell lung cancer patients who are elderly and/or have poor performance status Crit Rev Oncol Hematol 2011 76 1 53 60 19939700 10.1016/j.critrevonc.2009.10.005

[B6] Nguyen LN Effectiveness of accelerated radiotherapy for patients with inoperable non-small cell lung cancer (NSCLC) and borderline prognostic factors without distant metastasis: a retrospective review Int J Radiat Oncol Biol Phys 1999 44 5 1053 1056 10.1016/S0360-3016(99)00130-3 10421538

[B7] Fine JP Gray RJ A proportional hazards model for the subdistribution of a competing risk J Am Stat Assoc 1999 94 446 496 509 10.2307/2670170

[B8] Gray RJ A class of K-sample tests for comparing the cumulative incidence of a competing risk Ann Stat 1988 16 3 1141 1154 10.1214/aos/1176350951

[B9] Slotman BJ Hypofractionated radiation therapy in unresectable stage III non-small cell lung cancer Cancer 1993 72 6 1885 1893 10.1002/1097-0142(19930915)72:6<1885::AID-CNCR2820720616>3.0.CO;2-7 8395967

[B10] Kepka L Tyc-Szczepaniak D Bujko K Dose-per-fraction escalation of accelerated hypofractionated three-dimensional conformal radiotherapy in locally advanced non-small cell lung cancer J Thorac Oncol 2009 4 7 853 862 10.1097/JTO.0b013e3181a97dda 19487965

[B11] Cox JD Interruptions of high-dose radiation therapy decrease long-term survival of favorable patients with unresectable non-small cell carcinoma of the lung: analysis of 1244 cases from 3 Radiation Therapy Oncology Group (RTOG) trials Int J Radiat Oncol Biol Phys 1993 27 3 493 498 10.1016/0360-3016(93)90371-2 8226140

[B12] Machtay M Effect of overall treatment time on outcomes after concurrent chemoradiation for locally advanced non-small-cell lung carcinoma: analysis of the Radiation Therapy Oncology Group (RTOG) experience Int J Radiat Oncol Biol Phys 2005 63 3 667 671 10.1016/j.ijrobp.2005.03.037 15927409

[B13] Abratt RP Bogart JA Hunter A Hypofractionated irradiation for non-small cell lung cancer Lung Cancer 2002 36 3 225 233 10.1016/S0169-5002(02)00020-X 12009230

